# Enhancing EFL reading proficiency through mediated learning: Impacts on cognitive and metacognitive strategies

**DOI:** 10.1371/journal.pone.0337355

**Published:** 2026-04-16

**Authors:** Sisay Bezabih, Abiy Yigzaw, Birhanu Simegne, Dereje Assefa, Dawit Dibekulu

**Affiliations:** 1 Department of Language Education, School of Teacher Education, College of Education, Bahir Dar University, Bahir Dar, Ethiopia; 2 Department of English Language and Literature, Faculty of Humanities, Bahir Dar University, Bahir Dar, Ethiopia; Chengdu Normal University, CHINA

## Abstract

This study explored the impact of teacher educators’ mediation on the development of cognitive and metacognitive strategies to foster autonomous learning in English as a Foreign Language (EFL) reading skills among freshman pre-service teacher trainees. Additionally, it investigated how these strategies influenced reading performance. A mixed-methods quasi-experimental design was conducted at Kotebe University of Education, Addis Ababa, Ethiopia, with 80 first-year social science students randomly assigned to experimental (n = 40) and control (n = 40) groups. The experimental group received targeted mediation from an experienced English instructor, while the control group underwent traditional instruction. Data were gathered via reading proficiency tests, questionnaires, and classroom observations, analyzed using descriptive statistics (means, standard deviations, percentages), inferential statistics (t-tests), and thematic analysis for qualitative insights. Findings revealed that mediation significantly enhanced the experimental group’s use of cognitive and metacognitive strategies, promoting self-regulation and independent learning in EFL reading. The experimental group outperformed the control group in reading proficiency tests, with Cohen’s d indicating a large effect size. The study underscores the efficacy of mediated teaching in developing cognitive and metacognitive skills, fostering learner autonomy, and boosting academic success. It recommends that EFL instructors adopt mediation strategies to enhance students’ learning strategies and reading performance.

## 1. Introduction

English is a cornerstone of global communication, critical for education, business, and science [1,2]. In Ethiopia, English serves as the medium of instruction from primary through higher education, reflecting its critical importance in equipping learners with essential academic and professional language skills [3,4]. The Ethiopian Education and Training Policy mandates English instruction from grade one and its use as the primary medium in secondary schools and universities [3].

To align with global educational trends, Ethiopia has adopted a student-centered approach to English language instruction, moving away from traditional teacher-centered methods [4]. This approach emphasizes active learner participation, positioning teachers as facilitators who guide rather than dictate knowledge [5,6]. Such learner-centered environments foster collaboration between teachers and students, promoting decision-making skills and empowering learners to take ownership of their educational processes, a key tenet of learner autonomy [7,8].

Learner autonomy in English as a Foreign Language (EFL) reading relies on the development of cognitive and metacognitive strategies, including reasoning, analyzing, note-taking, summarizing, synthesizing, outlining, planning, monitoring, and evaluating [9]. Teachers play a crucial mediating role in cultivating these strategies, guiding students toward self-regulated learning and enhanced language performance [10,11]. For freshman pre-service teachers in Ethiopia and globally, mastering these strategies is vital for fostering autonomy and achieving academic success in EFL reading, while also preparing them to model such skills in their future teaching careers.

This study investigates the impact of teacher educators’ mediation on the development of cognitive and metacognitive strategies in EFL reading among freshman pre-service teachers in Ethiopia. By exploring how structured mediation enhances learner autonomy and reading proficiency, it aims to inform improved teaching practices and contribute to the effective implementation of Ethiopia’s EFL education policies.

## 2. Statement of the problem

Despite Ethiopia’s commitment to learner-centered education, many English language instructors continue to employ traditional, teacher-centered methods, which impede student engagement and autonomy [12,13]. Observations at Kotebe University of Education indicate that pre-service teacher trainees heavily rely on instructor-led guidance, constraining their opportunities for independent learning. Teacher educators have voiced concerns about this dependency, underscoring the need for greater awareness of their mediational role in cultivating learner autonomy.

Prior research in Ethiopia highlights a persistent gap in implementing strategies to promote learner autonomy. While some trainees exhibit awareness of cognitive strategies, their understanding and application of metacognitive strategies remain limited [14,15]. Furthermore, there is a significant lack of studies exploring the impact of teacher mediation on developing cognitive and metacognitive strategies to enhance autonomy in EFL reading within the Ethiopian context. In contrast, global research demonstrates the efficacy of explicit instruction and teacher mediation in fostering these strategies. For example, Aghaie and Zhang [16] found that Iranian EFL students receiving explicit metacognitive strategy instruction improved their reading comprehension and autonomy. Similarly, Guo [17] reported enhanced metacognitive awareness and independent learning behaviors among Chinese EFL learners through teacher mediation. Another study [18,11] showed that mediated strategy training significantly improved academic self-regulation and reading performance. However, these insights have not been adequately applied to Ethiopia’s EFL reading classrooms, an under-researched area.

This study addresses these gaps by examining how teacher educators’ mediation enhances cognitive and metacognitive strategies to promote autonomous EFL reading among Ethiopian pre-service teachers. By investigating the effects of structured mediation on learner autonomy and reading proficiency, it aims to contribute to improved teaching practices and policy implementation.

## 3. Literature review

### 3.1. Theoretical concepts of learner autonomy

Learner autonomy is a crucial aspect of language learning, as it empowers students to take control of their own learning process [19]. This entails learners being responsible for setting objectives, defining content and progression, and employing reflective and analytical skills to plan, monitor, and evaluate their learning effectively. Scholars like [20] and [2] state that learner autonomy encourages learners to develop their critical reflection and decision-making skills, in choosing learning goals, planning and organizing tasks, and evaluating their progress independently. In the context of EFL reading, autonomous learners can set their own learning goals, select appropriate strategies, monitor their progress, and evaluate their learning outcomes [21]. The development of cognitive and metacognitive strategies is central to fostering learner autonomy, as these strategies enable students to actively engage, take control, and regulate their own learning [22].

### 3.2. Cognitive and Metacognitive Strategies to Foster Learner Autonomy

The key to fostering learner autonomy lies in the teacher’s ability to help students develop both cognitive and metacognitive strategies. Cognitive strategies such as reasoning, analyzing, resourcing, note-taking, summarizing, synthesizing, and outlining enable learners to engage deeply with tasks, promoting more efficient learning [23,24]. These strategies help learners manipulate and process information in ways that improve comprehension and retention. Teachers play a crucial role in guiding learners to use these strategies effectively by modeling their application and encouraging their use in naturalistic settings [25]. Additionally, metacognitive strategies such as planning, monitoring, and evaluating progress allow learners to regulate and take control of their learning [24,26]. By guiding students to self-assess and adjust their approaches, teachers equip them with the tools necessary for self-directed learning, a fundamental aspect of learner autonomy [6,27].

Traditionally, teachers often serve as the primary source of knowledge, controlling both the content and delivery of teaching-learning [8]. However, to promote learner autonomy, the teacher’s role shifts towards facilitating and guiding students to take ownership of their learning [22,28]. Teachers support students by encouraging them to set learning objectives, monitor their progress, select appropriate materials, and evaluate their outcomes [29,28]. This facilitative role does not diminish the teacher’s importance; rather, it emphasizes the teacher as a mediator who fosters independence by gradually transferring responsibility to learners. By providing appropriate guidance, inspiration, feedback, and other forms of support, skillful mediators enable learners to employ cognitive and metacognitive strategies to self-regulate their learning and become self-directed learners [30]. Moreover, developing and employing these autonomous learning strategies (cognitive and metacognitive strategies) is also crucial in enhancing language performance and achieving better academic outcomes [31,32]

Teachers, therefore, act as mediators who facilitate learners’ cognitive and metacognitive development through intervention/mediation/ [10]. In this study, the term “mediation” is used synonymously with “intervention” to describe the appropriate roles teachers play in the development of learner autonomy in the context of English as a foreign language (EFL) learning of reading skills.

### 3.3. Teacher Mediation (Mediated Learning Experience (MLE)) in Developing Autonomy

The concept of Mediated Learning Experiences (MLE) has been a cornerstone of educational theory, particularly in the context of cognitive development and learner autonomy. Rooted in [28] sociocultural theory, MLE emphasizes the importance of social interaction and guidance from more knowledgeable individuals such as teachers or more capable peers, in fostering cognitive growth. Vygotsky also suggests that mediation/MLE/ refers to the role played by other significant people(teachers) in the learners’ lives who enhance their learning by selecting and shaping the learning experiences presented to them. The Zone of Proximal Development (ZPD), a key element of this theory, represents the growth potential that learners can achieve with appropriate support [11]. In relation to this, Vygotsky explains ZPD as the distance between the level of development indicated by the child’s/learner’s capacity to solve problems independently and the level of potential development indicated by his/her capacity to solve problems with the guidance and collaboration of other significant or competent persons(Teachers) [33]. Building on Vygotsky’s work, [34] expanded the concept of mediation, demonstrating its practical applications in educational settings and developing a comprehensive framework for MLE.

MLE emphasizes the role of a mediator who facilitates learning by making the environment more accessible and meaningful for the learner [34]. In the context of language learning, the teacher acts as the mediator, guiding students through the complexities of language acquisition and helping them develop cognitive and metacognitive strategies [35]. [36] identify several key parameters of MLE, such as intentionality and reciprocity, transcendence, meaning, regulation and control of behavior, goal-seeking and setting, and fostering a sense of competence, all of which work together to enhance cognitive functioning and enable structural cognitive modification (SCM).

Research has shown that effective mediation not only facilitates immediate learning but also equips learners with strategies for future independent problem-solving [18]. This aligns with Feuerstein’s emphasis on the role of mediators in selecting and shaping learning experiences to promote cognitive modifiability [34]. Moreover, [37] has demonstrated that intentional and reciprocal interactions between mediators and learners significantly enhance cognitive functions, particularly when combined with transcendence: the application of learning beyond immediate contexts. The practical implementation of mediation theory in educational settings has been the subject of numerous studies, with findings suggesting that mediation strategies, such as promoting intentionality and reciprocity, fostering transcendence, and emphasizing meaning, contribute to improved student engagement and academic performance [38,39]. Similarly, [39] noted that mediating behaviors related to self-regulation and goal-setting fostered greater learner autonomy and self-directed learning. These findings highlight the transformative potential of mediated learning experiences to transform educational practices, moving beyond traditional information dissemination to fostering critical thinking, problem-solving skills, and learner independence [11,34].

### 3.4. Reading Skills in EFL Context

Reading stands as a vital language skill, facilitating not only language proficiency but also comprehension of various subject areas [35,40,41]. By engaging with texts, learners enhance their thinking abilities, reflect on the content, and acquire knowledge [40,41]. Moreover, reading skills play a pivotal role in both academic and real-life lifelong learning settings, particularly in EFL contexts where students have limited exposure to the language [42]. Reading extends beyond mere information absorption; it demands a cognitive process that involves comprehending the writer’s perspective and intentions [43].

Viewing reading as an interactive, meaning-making, and problem-solving process, learners need to employ deliberate strategies and possess language proficiency, background knowledge, and topic-specific understanding [44]. By utilizing effective strategies, learners can enhance their reading comprehension skills, making the learning process more efficient, enjoyable, and independent [25]. Successful comprehension hinges on the interaction between decoding words, phrases, and sentences, understanding the text’s message, relating it to prior knowledge, and making logical inferences [45]. Consequently, English language teachers should be equipped with effective methods for teaching reading skills, enabling learners to develop cognitive and metacognitive strategies in learning reading skills autonomously within the classroom setting.

### 3.5. Conceptual framework and research gaps

**Fig 1:**
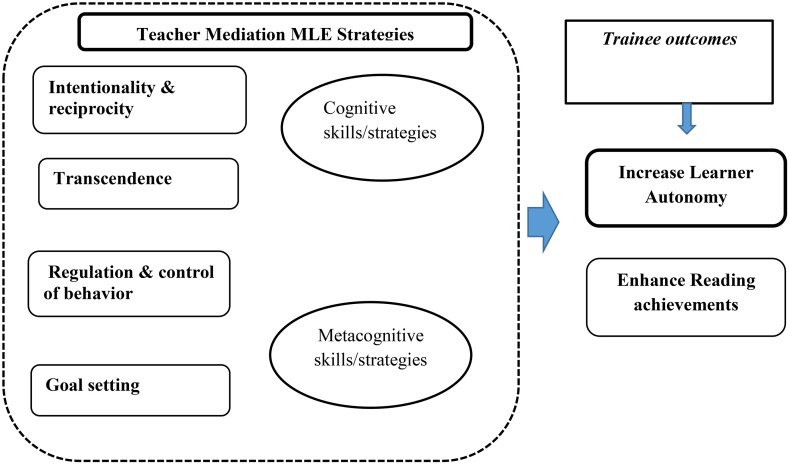
The conceptual framework.

This framework in Fig 1 below shows, the selected strategies of the Mediated Learning Experience (MLE) (such as intentionality and reciprocity, transcendence, regulation and control of behavior, and goal-setting) fit into the framework of two large categories of autonomous learning strategies, such as cognitive, and metacognitive, on which the data analysis in this study is based. *Cognitive strategies* are promoted through the mediation of transcendence (such as critical thinking like understanding, inferring, analyzing, synthesizing, organizing, evaluating, etc.). *Metacognitive strategies* also encompass mediation of intentionality and reciprocity (self-reflective), regulation and control of behavior (monitoring, evaluating and adjusting own learning), and goal setting (seeking, planning, and achieving). Besides, an experienced person (a mediator or teacher) scaffolds students to move from their actual level of development to their potential level of learner autonomy by mediating the learning strategies pertinent to autonomous learning with the appropriate assistance of the teacher’s mediation/intervention. The trainees’ autonomous engagement is expected to result in their high-level of reading achievement.

The conceptual framework guiding this study (Fig 1) synthesizes three critical dimensions of language learning: Feuerstein’s Mediated Learning Experience (MLE) principles, cognitive and metacognitive strategy development, and EFL reading skill acquisition. This integrative approach reveals several significant gaps in current research that this study aims to address. First, while the importance of teacher mediation in language learning is well-established [11,18], there remains a paucity of research examining how mediation functions specifically in preservice teacher education contexts. This gap is particularly noteworthy given that preservice teachers must simultaneously develop their own autonomous learning strategies while preparing to facilitate such strategies in their future students. Second, although MLE principles have been applied broadly in educational settings [34,37], few studies have investigated their specific relationship with reading autonomy development in EFL contexts. Third, while learner autonomy and reading strategies have been studied in various settings, there is a distinct lack of empirical evidence from Ethiopian EFL contexts, where unique educational policies and linguistic landscapes may influence learning outcomes [3,4,46]. This study seeks to address these interconnected gaps by investigating how teacher educators’ mediation impacts the development of cognitive and metacognitive reading strategies among Ethiopian preservice teachers, thereby contributing new insights to both theoretical understanding and practical application in teacher education programs.

The practical implementation of mediation strategies, such as intentionality, reciprocity, transcendence, and meaning, has been shown to enhance student engagement and academic performance [38,39]. For instance, Guo [17] demonstrated that teacher mediation significantly improved Chinese EFL learners’ metacognitive awareness and independent learning behaviors. Similarly, mediated strategy training has been linked to improved self-regulation and reading comprehension in various EFL contexts [11,18,23]. However, these studies primarily focus on general EFL learners, with limited attention to pre-service teacher trainees, who must develop both their own learning autonomy and the ability to foster it in future students. Moreover, while these global findings highlight the efficacy of mediation, there is a notable lack of research exploring its application in the Ethiopian EFL context, particularly in developing cognitive and metacognitive strategies for reading skills among pre-service teachers. In Ethiopia, prior studies [14,15] indicate limited use of metacognitive strategies among learners and a reliance on traditional teaching methods, underscoring a gap in understanding how teacher mediation can foster learner autonomy in EFL reading classrooms.

Given these gaps, this study seeks to address the following research questions, which are derived from the identified deficiencies in the literature and tailored to the Ethiopian context of pre-service teacher education:

### Research questions

1. Does teacher educators’ mediation significantly enhance pre-service trainees’ use of cognitive and metacognitive strategies for autonomous EFL reading compared to a control group receiving traditional instruction?
2. Does teacher educators’ mediation improve trainees’ reading proficiency through enhanced use of cognitive and metacognitive strategies?

## 4. Methodology

### 4.1. Research design

This study employed a mixed-methods quasi-experimental design to investigate the impact of teacher educators’ mediation on the development of cognitive and metacognitive strategies for fostering autonomous EFL reading skills among pre-service teachers at Kotebe University of Education, Addis Ababa, Ethiopia. The design integrated quantitative data collection through reading proficiency tests, questionnaires, and structured observation checklists, complemented by qualitative data from field notes capturing contextual insights during classroom observations. This mixed-methods approach enabled a comprehensive examination of the independent variable (teacher mediation strategies) and its effects on dependent variables (trainees’ perceived use of cognitive and metacognitive strategies and reading proficiency outcomes). The quasi-experimental design was chosen for its suitability in intact classroom settings, common in Ethiopian higher education, where students are pre-assigned to groups, minimizing disruption to regular teaching processes while maintaining ecological validity [47,48].

### 4.2. Research setting

This study was conducted at Kotebe University of Education (KUE) in Addis Ababa, Ethiopia, during the first semester of the 2023/24 academic year (December 5, 2023, to March 22, 2024). KUE, formerly known as Kotebe College of Teacher Education, has a rich history spanning over 65 years, originating as a teacher training program within Haile Selassie I University in 1959. It evolved into Kotebe University College in 2014 and was elevated to university status in 2016, becoming Kotebe Metropolitan University before being re-designated as KUE in 2022 under Proclamation No. 1263/2014. As a leading institution for teacher education in Ethiopia, KUE focuses on producing qualified educators through undergraduate and postgraduate programs, aligning with the Ministry of Education’s goal of enhancing educational quality nationwide [49]. With approximately 9,200 students and 354 academic staff, KUE’s College of Educational Sciences is particularly relevant for this study, as it trains pre-service teachers in learner-centered pedagogies, including EFL instruction.

KUE was purposively selected as the research setting due to its prominence in teacher education, its focus on EFL training for pre-service teachers, and its urban location in Addis Ababa, which ensures access to diverse student populations and robust academic resources [47,50]. The university’s commitment to learner-centered education, as evidenced by its curriculum reforms and research initiatives, makes it an ideal context for investigating teacher mediation’s impact on cognitive and metacognitive strategies in EFL reading. Additionally, KUE’s pre-assigned classroom groups align with the quasi-experimental design’s requirements, minimizing disruption to regular teaching processes [47,48]. The study spanned 13 weeks, including one week for pre-testing and questionnaires, one week for post-testing and questionnaires, and one week for training the teacher-mediator, ensuring a structured timeline for data collection and intervention implementation.

### 4.3. Sample and sampling techniques

The study population consisted of 598 first-year pre-service teachers (318 male, 280 female) enrolled in Kotebe University of Education’s (KUE) regular degree program during the 2023/24 academic year. Admission was based on standardized criteria, including high school transcripts and Ethiopian General Secondary Education Certificate Examination (EGSECE) results. A multi-stage sampling approach was employed to select participants. Initially, trainees were stratified by academic stream to account for disciplinary differences. Participants were first-year pre-service teachers aged 18–21, enrolled in the Communicative English Language Skills I (FLEn 1011) course, with intermediate English proficiency based on EGSECE results. Added Students outside the 18–21 age range or not enrolled in FLEn 1011 were excluded to ensure homogeneity. The two intact social science classes (Sections 1 and 3, n = 40 each) were comparable in class size, curriculum, and scheduling, with no significant differences in student demographics (e.g., age, gender) or prior academic performance, as confirmed by pre-intervention t-test results (Table 3, p = .939) Two intact social science classes (n = 80) enrolled in the Communicative English Language Skills I (FLEn 1011) course were purposively selected, as social science students typically exhibit stronger engagement with language learning compared to natural science students, ensuring homogeneity in English proficiency [47,50]. The sample comprised 80 trainees aged 18–21, with Section 1 (n = 40; 19 female, 21 male) and Section 3 (n = 40; 18 female, 22 male) selected to maintain natural classroom dynamics while controlling for variables such as age, academic standing, and English proficiency. These sections were randomly assigned to experimental (Section 1) and control (Section 3) groups via a lottery system, ensuring group equivalence and minimizing selection bias to enhance internal validity [47,48]. Additionally, two experienced English instructors were selected using snowball sampling, a method suitable for identifying knowledgeable informants in specialized educational contexts [50]. The sample size of 80 trainees was determined to meet the requirements for quasi-experimental designs, balancing statistical power to detect medium effect sizes with practical constraints of classroom-based research. This multi-stage sampling strategy ensured methodological rigor and ecological validity, making the findings relevant to Ethiopia’s teacher education context.

#### 4.3.1. Instructor profiles and training.

Two experienced English instructors were selected using snowball sampling. Both had over 10 years of EFL teaching experience at Kotebe University of Education and held Master’s degrees in Teaching English as a Foreign Language (TEFL). The teacher-mediator for the experimental group received 8 hours of training on Mediated Learning Experience (MLE) strategies, focusing on intentionality, reciprocity, transcendence, and goal-setting, delivered in Week 2 of the study. The control group instructor followed the standard Communicative English Language Skills I curriculum without MLE training. Intervention fidelity was ensured through weekly reviews of lesson plans and observation checklists to confirm consistent application of MLE strategies in the experimental group. This ensured that the intervention was delivered consistently and that instructor differences were minimized to strengthen the study’s internal validity.

### 4.4. Data collection instruments

To collect comprehensive data from participants, this study employed multiple instruments, including reading proficiency tests, questionnaires, and classroom observations. These tools were designed to complement each other, ensuring triangulation of quantitative and qualitative data to enhance the validity and reliability of the findings.

#### 4.4.1. Reading proficiency tests.

To assess the reading proficiency and knowledge of pre-service teacher trainees before and after the intervention, two parallel versions of reading proficiency tests were adapted from the ‘Ereading’ website [51]. The ‘Ereading’ tests were selected due to their alignment with the study’s objectives, offering a robust framework for evaluating EFL reading comprehension skills relevant to the Communicative English Language Skills I (FLEn 1011) course. These tests are recognized as standardized tools, widely used in educational research for their reliability, validity, and comprehensive coverage of reading skills, as evidenced by their design to assess diverse comprehension abilities across various proficiency levels [51, 52]. Their adaptability allowed the researcher to tailor items to match the trainees’ intermediate English proficiency levels, ensuring cultural and contextual relevance for Ethiopian pre-service teachers [52]. The tests were modified by incorporating tasks aligned with the course module’s objectives, such as predicting content, inferring word meanings, skimming, scanning, and drawing conclusions [53]. Each test comprised 25 multiple-choice, true/false, and matching items, assessing key reading comprehension aspects, including making predictions, inferring meanings from context, skimming for gist, scanning for specific information, making inferences, and drawing conclusions beyond explicit text content. One version was administered as the pre-intervention assessment to both the experimental group (EG) and control group (CG) simultaneously in separate locations, while the second version served as the post-intervention assessment at the semester’s end. Each test had a 45-minute time limit to ensure consistency and manageability.

#### 4.4.2. Questionnaires.

This study utilized a questionnaire as the primary instrument to quantitatively evaluate the impact of teacher educators’ mediation on the development of cognitive and metacognitive strategies for autonomous EFL reading among pre-service teachers. The questionnaire was structured in two sections: the first collected trainees’ demographic information, while the second comprised three parts. The first two parts assessed trainees’ perceptions of the importance of mediation parameters and the teacher-mediator’s application of these strategies, using a five-point Likert scale (1 = strongly disagree, 5 = strongly agree). The third part examined the perceived use of autonomous learning strategies, consisting of 36 items—12 targeting cognitive strategies and 24 addressing metacognitive strategies, with three reverse-coded items to reduce response bias. Items were adapted from established studies [24,25,54,55,56] and refined through expert review to ensure alignment with the study’s objectives and cultural context.

The 36-item questionnaire, adapted from O’Malley and Chamot [24] and Oxford [25], assessed perceived use of cognitive (12 items) and metacognitive (24 items) strategies. Reliability was confirmed with Cronbach’s alpha (Cognitive strategies: α = 0.81; Metacognitive strategies: α = 0.85) and McDonald’s omega (Cognitive strategies: ω = 0.83; Metacognitive strategies: ω = 0.87). Confirmatory Factor Analysis (CFA) on the pilot (n = 26) and main (n = 80) samples showed acceptable model fit (CFI = 0.92, TLI = 0.90, RMSEA = 0.06 for the main sample). Sampling adequacy was verified with KMO = 0.79 and Bartlett’s test p < .001. All items had item–total correlations > 0.3, with three reverse-coded items retained after pilot testing. No items were deleted, as all met reliability and validity thresholds during expert review. The complete 36-item questionnaire, including operational definitions and subdimension structure (cognitive: e.g., summarizing, analyzing; metacognitive: e.g., planning, monitoring, evaluating).

To enhance accessibility for Ethiopian trainees, the questionnaire was developed in English and translated into Amharic, with bilingual instructions provided during administration to ensure clarity. A pilot study with 26 teacher trainees at Kotebe University of Education identified and resolved issues related to item clarity and comprehension. Reliability was confirmed using SPSS version 25, yielding Cronbach’s alpha values above 0.70 for all sections, indicating strong internal consistency [57]. Validity was further strengthened through iterative expert reviews by TEFL specialists, resulting in revisions to item wording and structure to ensure relevance and precision.

#### 4.4.3. Classroom Observation.

This study utilized classroom observation to examine participants’ interactions during reading tasks in the experimental group and to evaluate the teacher-mediator’s role in implementing mediation strategies. As Bell [58] notes, observation captures authentic classroom practices that may not be fully reflected in other data collection methods, providing direct insights into intervention dynamics. This method was selected to assess the application of mediation strategies designed to foster autonomous learning in EFL reading, aligning with the study’s focus on cognitive and metacognitive strategy development [11]. An observation checklist, adapted from O’Malley and Chamot [24] and Vygotsky [11], was used to systematically evaluate the teacher-mediator’s use of strategies such as intentionality, reciprocity, and transcendence.Thirteen classroom observations were conducted over 13 weeks of reading skills sessions, with a co-observer assessing the effectiveness of the mediation strategies. Field notes were also recorded to capture contextual details, including classroom dynamics, student engagement, and the overall learning environment, enhancing the qualitative depth of the data. Thirteen classroom observations were conducted with two observers. Inter-rater reliability was assessed using Cohen’s kappa, yielding an average κ = 0.82 (substantial agreement) across seven cognitive strategy items (e.g., “Connecting reading materials to prior knowledge”) and 13 metacognitive strategy items (e.g., “Setting learning objectives”). Observer ratings were averaged, with discrepancies resolved through discussion to ensure consistency. The observation checklist, including seven cognitive strategy items and 13 metacognitive strategy items, with operational definitions and scoring rules (Yes = 1, No = 0), is provided in Appendix A.

#### 4.4.4. Validity and reliability.

4.4.4.1. ***Validity*:** The study employed multiple strategies to ensure the validity of research instruments. For the reading proficiency tests, content and construct validity were established through rigorous expert review. A panel comprising a language assessment specialist, two TEFL experts, and a measurement and evaluation specialist examined the test items, leading to the revision and deletion of certain questions to better align with the study’s objectives. The questionnaires underwent similar scrutiny by two supervisors and two PhD candidates in TEFL, resulting in modifications to item wording and structure.

The questionnaire items were carefully adapted from established instruments [54,24,25,55, 56] and specifically tailored to assess EFL reading skills. To enhance comprehension, all items were translated into Amharic by a qualified translator and further refined with input from TEFL experts. The instruments were pilot-tested with 26 teacher trainees participating in a summer program at Kotebe University of Education prior to the main study. This process identified and eliminated various issues including double-barreled questions, ambiguous wording, unclear instructions, and inadequate response scales.

4.4.4.2. ***Reliability*:** The reliability of research instruments was assessed through internal consistency measures. Pilot testing yielded strong Cronbach’s alpha coefficients for all instruments: the 25-item reading tests demonstrated excellent reliability (α = 0.872 and 0.823), while the 36-item language learning strategies questionnaire showed high consistency (α = 0.839). The 10-item scales measuring trainees’ perceptions of mediation principles also achieved acceptable reliability (α = 0.837 for importance ratings and α = 0.743 for mediator’s use). Following [57] threshold, all instruments were deemed reliable as their alpha values exceeded 0.70. These results confirm that the research tools consistently measured their intended constructs, supporting the credibility of the study’s findings.

### 4.5. Teaching materials

For both the experimental and control groups, reading skills teaching materials were sourced from the “Communicative English Language Skills – I” (FLEn 1011) course module, tailored for Ethiopian university students [53]. This module emphasized developing listening and reading skills while integrating speaking and writing activities. It comprised five reading texts with pre-reading, while-reading, and post-reading activities, representing about 31% of the module.

The reading section was allocated 13 class sessions of 50 minutes each, equating to one-third of the total course duration. While the control group received conventional instruction using the standard materials, the experimental group was taught modified reading activities designed using mediation strategies. These modifications included new questions and guided activities to scaffold the development of cognitive and metacognitive strategies.

In the pre-reading phase, activities were designed to activate prior knowledge and set learning goals through brainstorming and visual aids. During the while-reading stage, trainees engaged with activities such as inferring, analyzing, organizing, synthesizing, etc. to guid and monitor comprehension while reading texts. Post-reading activities facilitated reflection, summarization, and real-life application of learning, encouraging self-assessment and goal-setting. Overall, the experimental materials aimed to align with constructivist principles and promote learner autonomy within a sociocultural framework [59].

### 4.6. Research procedure

This study adopted a multi-phased approach to investigate the impact of teacher educators’ mediation on cognitive and metacognitive strategy development for autonomous EFL reading among pre-service teachers at Kotebe University of Education (KUE) [11,39]. The study spanned 13 weeks (December 5, 2023, to March 22, 2024), encompassing preparation, intervention, and data collection phases.

The preparation phase involved adapting instructional reading materials to incorporate Mediated Learning Experience (MLE) strategies, modifying assessment instruments, and developing a manual to guide teacher-mediators in fostering cognitive and metacognitive strategies [26,30]. Training sessions (8 hours per week) were conducted in Week 2 to prepare the teacher-mediator and a reserve trainer on MLE strategies and materials. Ethical approval was obtained from KUE’s college dean and Department Head of English, with oral consent secured from all participants (teacher educators and trainees) after clear explanations of the study’s objectives, procedures, and voluntary participation rights. As a low-risk educational study involving standard classroom activities, oral consent was deemed sufficient, aligning with ethical guidelines [60, 61]. Data were anonymized, participation did not affect academic standing, and participants were debriefed on outcomes to ensure ethical compliance.

The intervention spanned 10 weeks (December 11, 2023, to March 15, 2024), with the experimental group (EG) receiving 13 reading lessons incorporating MLE strategies, while the control group (CG) followed conventional instruction. The teacher-mediator employed strategies to scaffold cognitive and metacognitive skills, fostering learner autonomy [11]. Pre-reading activities included activating prior knowledge, goal-setting, and negotiating task purposes through brainstorming and visual aids. During reading, the mediator facilitated vocabulary highlighting, context clue provision, predictions, reflections, and comprehension monitoring, encouraging cognitive strategies (e.g., inferring, analyzing, synthesizing) and metacognitive strategies (e.g., self-monitoring). Post-reading activities involved reflection, summarization, opinion-sharing, and self-assessment to apply learning to real-life contexts, aligning with constructivist principles [59]. Pre-intervention data collection (Week 1) involved administering reading proficiency tests and questionnaires to both groups to establish baseline homogeneity.

Post-intervention data collection (March 18–22, 2024) used a parallel reading proficiency test of similar difficulty and questionnaires to assess changes in reading skills and perceptions of mediation strategies, focusing on cognitive and metacognitive strategy development and learner autonomy.

Ethical approval was obtained from the Bahir Dar University, College of Education, School of Teacher Education, Department of Language Education Research Ethics Committee (Approval No. Lang/08/2016/2023).Verbal consent was obtained from all participants (teacher educators and trainees) due to the low-risk nature of the study, which involved standard classroom activities, aligning with British Educational Research Association (BERA) ethical guidelines [60]. Minors were excluded, as all participants were aged 18–21, confirmed by university enrollment records.

Intervention fidelity was ensured through weekly reviews of lesson plans and observation checklists, conducted by the researcher and a co-observer, to confirm that the teacher-mediator consistently applied MLE strategies (e.g., intentionality, reciprocity, transcendence) in the experimental group.

### 4.7. Data analysis

This study employed a mixed-methods analytical approach to examine quantitative and qualitative data, with techniques selected to align with the research objectives of investigating teacher educators’ mediation on cognitive and metacognitive strategy development for autonomous EFL reading. Quantitative data from reading proficiency tests and questionnaires were analyzed using SPSS version 25. Normality was confirmed through the Kolmogorov-Smirnov test (p > .05 for all groups), skewness (−1 < values < 1), and kurtosis (−1 < values < 1), justifying the use of parametric tests [62]. Independent samples t-tests compared means between the experimental (EG) and control (CG) groups, while paired samples t-tests assessed within-group changes from pre- to post-intervention, ensuring robust evaluation of group differences and progress over time [27, 57]. Cohen’s d effect sizes were calculated to determine practical significance, using benchmarks of 0.2 (small), 0.5 (moderate), and 0.8 (large) [57]. Descriptive statistics, including means (M) and standard deviations (SD), complemented inferential analyses to provide a comprehensive overview of results.

Qualitative data from classroom observations of the EG were collected via checklists and field notes. Checklist responses were quantified as percentages and thematically categorized based on mediation strategies and learner autonomy variables, while field notes were analyzed thematically to capture classroom dynamics and contextual insights. These qualitative findings served as complementary evidence, triangulating the quantitative results to enhance the study’s validity and depth.

## 5. Results and discussion

This section presents the findings of the mixed-methods study, addressing the research questions on the impact of teacher educators’ mediation on cognitive and metacognitive strategy development for autonomous EFL reading among pre-service teachers. The results are organized into quantitative and qualitative analyses, providing a comprehensive evaluation of the intervention’s effectiveness.

### 5.1. Analysis of quantitative data

The quantitative data analysis examines reading proficiency test scores and questionnaire responses to determine the effects of mediation strategies on trainees’ cognitive and metacognitive strategy use and reading performance. Statistical tests were employed to compare the experimental group (EG) and control group (CG), with preliminary analyses ensuring data suitability for parametric testing.

#### 5.7.1. Test of normality.

To confirm the appropriateness of parametric statistical tests, the normality of reading proficiency test scores for both the Experimental Group (EG) and Control Group (CG) was assessed. The Shapiro-Wilk and Kolmogorov-Smirnov tests were conducted, with results presented in Table 1.

**Table 1:** Test of normality.

Tests and Groups	Kolmogorov-Smirnov			Shapiro-Wilk		
Trainees’ reading skills score	Statistic	Df	Sig.	Statistic	Df	Sig.
Pre-test(EG)	.091	40	.200*	.976	40	.558
Pre-test(CG)	.119	40	.158	.966	40	.264
Post-test(EG)	.101	40	.200*	.987	40	.924
Post-test(CG)	.085	40	.200*	.976	40	.531

*. This is a lower bound of the true significance.

a. Lilliefors Significance Correction

The Kolmogorov-Smirnov and Shapiro-Wilk tests yielded significance (Sig.) values above 0.05 for all groups, confirming that the reading proficiency test scores were normally distributed. Skewness (−1 < values < 1) and kurtosis (−1 < values < 1) values further supported normality, justifying the use of independent and paired samples t-tests for subsequent analyses [62].

#### 5.7.2. Descriptive statistical results of trainees’ reading skills tests scores.

This subsection presents the results of the reading proficiency tests for pre-service teacher trainees, providing insights into the impact of teacher educators’ mediation on EFL reading skills. Descriptive statistics for pre- and post-intervention reading test scores for the Experimental Group (EG) and Control Group (CG) are summarized in Table 2, highlighting performance differences between groups.

**Table 2:** Descriptive statistics.

Reading Skills Tests Scores	N	EG		CG	
		M	SD	M	SD
Pre-test	40	10.93	2.98	10.88	2.88
Post-test	40	15.68	3.53	11.20	3.51

***Key*** *: N = numbers of Participants, EG = Experimental Group, CG = Control Group, M = Mean, SD = Standard Deviation*

The pre-intervention reading scores showed comparable baseline performance, with the EG achieving a mean of 10.93 (SD = 2.98) and the CG a mean of 10.88 (SD = 2.88), indicating group equivalence prior to the intervention. Post-intervention, the EG demonstrated a substantial increase, with a mean score of 15.68 (SD = 3.53), compared to the CG’s mean of 11.20 (SD = 3.51). These results suggest that the mediation strategies employed in the EG significantly enhanced reading proficiency compared to the conventional instruction received by the CG, laying the groundwork for further statistical analysis of the intervention’s impact.

#### 5.7.3. Results of Trainees’ Reading skills Pre- and Post-test scores.

This subsection analyzes the pre- and post-intervention reading proficiency test scores to evaluate the impact of teacher educators’ mediation on EFL reading skills among pre-service teachers. The analysis begins with a pre-intervention comparison to confirm group equivalence, followed by subsequent analyses of post-intervention outcomes.

**5.7.3.1. *Pre-intervention independent sample t-test results in the pre-test of reading skills performance*:** The pre-intervention reading proficiency test established baseline equivalence in reading skills between the Experimental Group (EG) and Control Group (CG). As shown in Table 2 (see Section 5.1.2), the EG had a mean score of 10.93 (SD = 2.98), and the CG had a mean score of 10.88 (SD = 2.88), indicating comparable performance. An independent samples t-test was conducted to confirm this homogeneity.

The t-test results (Table 3) showed no significant difference in pre-intervention reading skills between the EG (M = 10.93, SD = 2.98) and CG (M = 10.88, SD = 2.88; t(78) =.076, p = .939, two-tailed). The mean difference was minimal (.050, 95% CI: −1.256 to 1.356), with a negligible effect size (Cohen’s d = .017). These findings confirm the groups’ equivalence in reading proficiency prior to the intervention, establishing a robust baseline for subsequent analyses.

**Table 3:** Independent samples t-test results of the Pre-intervention reading skills scores.

Trainees’ Reading Skills Score	Levene’s Test for Equality of Variances		t-test for Equality of Means					95% Confidence Interval of the Difference	
	F	Sig.	T	df	Sig. (2-tailed)	Mean Difference	Std. Error Difference	Lower	Upper
Equal variances assumed	.027	.869	.076	78	.939	.050	.656	−1.256	1.356
Equal variances not assumed			.076	77.91	.939	.050	.656	−1.256	1.356

5.7.3.2. ***Post-intervention paired sample t-test results of the post-test reading skills performanceP*:** Table 4 presents the paired-sample t-test and Cohen’s d results, which were computed to examine the differences between pre-test and post-test reading skills performance within the experimental (EG) and control groups (CG). The analysis also assessed the magnitude of the effect of teacher mediation.

**Table 4:** The results of the Paired Samples Test on EFL trainees’ Reading skills post – test score.

Group	N	Reading Skills Tests	Mean	Paired Differences							
				Mean	SD	Std. Error Mean	95% Confidence Interval of the Difference				
							Lower	Upper	t	df	Sig. (2-tailed)
EG	40	Pre-test	10.93	−4.750	1.997	.316	−5.389	−4.111	−15.05	39	.000
		Post-test	15.68								
CG	40	Pre-test	10.88	−.325	1.328	.210	−.750	.100	−1.548		−1.30
		Post-test	11.20								

The paired-sample t-test results revealed no statistically significant difference in the CG’s reading skills scores between the pre-test (M = 10.88, SD = 2.88) and post-test (M = 11.20, SD = 3.51), t(39) = −1.548, p > .05. The small mean difference (0.32) and trivial effect size (Cohen’s d = 0.10) indicate that no meaningful improvement occurred in the absence of teacher mediation. In contrast, the EG showed a statistically significant improvement in reading performance from the pre-test (M = 10.93, SD = 2.98) to the post-test (M = 15.68, SD = 3.53), t(39) = −15.05, p < .001. The mean difference of 4.75, accompanied by a large effect size (Cohen’s d = 1.45), demonstrates a strong and substantial impact of teacher mediation on enhancing the reading skills of EG participants.

#### 5.7.4. Results of Trainees’ Views on the importance and mediator’s use of Mediation Parameters.

In Table 5, a paired-sample-test and Cohen’s d tests were computed to investigate trainees’ reflections on the importance and mediator’s use of mediation parameters before and after the intervention within the experimental group.

**Table 5:** Paired Sample t-test results of participants’ (EG) views on the importance and mediator’s use of Mediation Parameters.

Group	Pair No.	Variables	N	M	SD	T	df	Sig(2-tailed)	Cohen’s d
EG	Pair1	Pre-importance	40	2.63	.382	−11.988	39	.000	2.53
		Post-importance	40	3.88	.586				
	Pair2	Pre-use	40	2.71	.409	−13.648	39	.000	2.94
		Post-use	40	3.91	.406				

A paired-sample t-test was conducted to examine teacher trainees’ reflections on the importance of mediation parameters and their perceptions of the mediator’s application of these principles. The results revealed a statistically significant improvement in trainees’ views on the importance of mediation parameters, with scores increasing from the pre-test (M = 2.63, SD = 0.38) to the post-test (M = 3.88, SD = 0.59), t(39) = −11.99, p < .001, Cohen’s d = 2.53. This finding indicates a large effect size.

Similarly, trainees’ perceptions of the mediator’s actual use of mediation principles also showed a significant improvement, rising from the pre-test (M = 2.71, SD = 0.41) to the post-test (M = 3.91, SD = 0.46), t(39) = −13.65, p < .001, Cohen’s d = 2.94. This result likewise indicates a very strong effect of the intervention.

#### 5.7.5. Results of Trainees’ Perceived Use of CS and MCS in Pre- and Post-intervention.

This section presents the findings related to trainees’ perceived use of cognitive strategies (CS) and metacognitive strategies (MCS) before and after the intervention. The analysis was carried out to evaluate the extent to which teacher mediation influenced participants’ self-reported employment of these strategies. Questionnaire results reflect trainees’ perceived use of cognitive and metacognitive strategies, while reading proficiency test scores provide objective evidence of performance improvement. A clarifying statement was added: “While self-reported strategy use indicates trainees’ perceptions of their strategic engagement, improvements in reading proficiency test scores provide objective evidence of enhanced performance attributable to mediation.” The results are organized into two parts: the pre-intervention outcomes, which provide baseline insights into the comparability of the experimental and control groups, and the post-intervention outcomes, which highlight the potential changes attributable to the intervention.

**5.7.5.1. Pre-intervention results of freshman pre-service trainees’ perceived use of CS and MCS:** The pre-intervention questionnaire was administered to establish baseline data and determine the homogeneity of the two intact groups (experimental group [EG] and control group [CG]) regarding their perceived use of cognitive strategies (CS) and metacognitive strategies (MCS) prior to the intervention.

As shown in Table 6, the mean scores of the two groups on both constructs were relatively close, with differences of 0.17 for CS and 0.08 for MCS, suggesting comparable levels of perceived strategy use before the intervention. To determine whether these observed differences were statistically significant, an independent-samples t-test was computed. The results are presented in Table 7.

**Table 6:** Pre-intervention descriptive statistics.

Variables	N	EG		CG	
		M	SD	M	SD
Pre-intervention					
CS	40	2.43	.772	2.26	.603
MCS	40	2.09	.307	2.01	.458

**Table 7:** Pre-intervention independent sample t-test.

CS/MCS	Equal variance	Levene’s Test for Equality of Variances		t-test for Equality of Means				
		F	Sig	T	Df	Sig(2-tailed)	Mean Difference	Std. Error Difference
CS	Assumed	2.162	.145	1.043	78	.300	.170	.163
MCS	Assumed	6.767	.011	0.846	78	.400	.075	.087

Results, in Table 7, proved that there was no statistically significant difference in CS mean scores for EG ((M = 2.43, SD = .772) and the CG (M = 2.26, SD = .603; t (78) = 1.043, p > .05, 2-tailed); and in MCS mean scores for EG (M = 2.09, SD = .307) and the CG (M = 2.01, SD = .458; t (78) =.846, p > .05, 2-tailed). Besides, Cohen’s d statistic for CS (0.23) and MCS (0.19) indicated a small effect size. The sig. (two-tailed) values of the constructs were greater than the cut-off alpha level,.05. Therefore, it was assumed that the trainees were homogenous before the intervention.

**5.7.5.2. Post-intervention results of freshman pre-service trainees’ perceived use of CS and MCS:** The analysis of the post-intervention mean scores indicated that the experimental group (EG) obtained higher scores than the control group (CG) on both cognitive strategies (CS) and metacognitive strategies (MCS). In addition, both groups showed higher mean scores at post-test compared to pre-test. The descriptive statistics for the two groups across pre- and post-intervention are presented in Table 8.

**Table 8:** Descriptive statistics of Pre- and post-intervention.

Variables	N	EG		CG	
Pre-intervention		M	SD	M	SD
CS	40	2.43	.772	2.26	.603
MCS	40	2.09	.307	2.01	.458
Post-intervention					
CS	40	3.54	.772	2.41	.619
MCS	40	3.33	.468	2.06	.418

As shown in Table 8, the EG’s mean scores for CS and MCS increased from pre-test (M = 2.43, SD = 0.77; M = 2.09, SD = 0.31) to post-test (M = 3.54, SD = 0.77; M = 3.33, SD = 0.47). The CG also showed slight increases, from pre-test (M = 2.26, SD = 0.60; M = 2.01, SD = 0.46) to post-test (M = 2.41, SD = 0.62; M = 2.06, SD = 0.42). To determine whether these differences were statistically significant, paired-sample t-tests with Cohen’s d were conducted. The results are summarized in Table 9.

**Table 9:** Paired Sample t-test results of participants’ (EG and CG) rating of perceived use of CS and.

Groups	Pair No.	Variables	N	M	SD	T	df	Sig(2-tailed)	Cohen’s d
EG	Pair1	Pre-CS	40	2.43	.772	−4.799	39	.000	1.44
		Post-CS	40	3.54	.772				
	Pair2	Pre-MCS	40	2.09	.307	−12.383	39	.000	3.15
		Post-MCS	40	3.33	.468				
CG	Pair3	Pre-CS	40	2.26	.603	−1.563	39	.126	0.23
		Post-CS	40	2.41	.619				
	Pair4	Pre-MCS	40	2.01	.459	−1.115	39	.272	0.11
		Post-MCS	40	2.06	.418				

The results in Table 9 show that the EG demonstrated statistically significant improvements in both CS (t(39) = −4.80, p < .001, Cohen’s d = 1.44) and MCS (t(39) = −12.38, p < .001, Cohen’s d = 3.15), with large effect sizes. In contrast, the CG did not exhibit statistically significant changes in either CS (t(39) = −1.56, p > .05, Cohen’s d = 0.23) or MCS (t(39) = −1.12, p > .05, Cohen’s d = 0.11).

These findings suggest that teacher mediation had a substantial and positive effect on trainees’ perceived use of both cognitive and metacognitive strategies in the experimental group, while no meaningful changes occurred in the control group.

The post-intervention comparison between the EG (N = 40) and CG (N = 40) indicated that the experimental group outperformed the control group on both dependent variables (CS and MCS). To determine whether these observed differences were statistically significant, independent-samples t-tests with Cohen’s d were conducted. The results are presented in Table 10.

**Table 10:** Post-intervention independent sample t-test.

CS/MCS	Groups	M	SD	N	t	Df	Sig(2-tailed)	Cohen’s d
CS	EG	3.54	.772	40	7.058	78	.000	1.58
	CG	2.41	.619	40				
MCS	EG	3.33	.468	40	12.817	78	.000	2.87
	CG	2.06	.418	40				

As shown in Table 10, the EG scored significantly higher than the CG in both CS (M = 3.54, SD = 0.77) and MCS (M = 3.33, SD = 0.47), compared to the CG’s scores in CS (M = 2.41, SD = 0.62) and MCS (M = 2.06, SD = 0.42). These differences were statistically significant for both CS (t(78) = 7.06, p < .001) and MCS (t(78) = 12.82, p < .001). Furthermore, the effect sizes were large (Cohen’s d = 1.58 for CS and 2.87 for MCS), providing strong evidence that teacher mediation substantially enhanced trainees’ perceived use of both cognitive and metacognitive strategies.

#### 5.7.6. Lesson observation results.

A total of 13 reading skills lessons were observed by the researcher in collaboration with a co-observer. The focus of the observation was to examine how frequently teacher trainees were facilitated or mediated through specific mediation strategies designed to enhance the development of cognitive and metacognitive strategies.

Cohen’s kappa was calculated for the 13 observation sessions, yielding an average κ = 0.82, indicating substantial agreement between observers for the seven cognitive strategy items and 13 metacognitive strategy items. Discrepancies were resolved through discussion, ensuring reliable data collection. The observation checklist, including items, operational definitions, and scoring rules to enhance transparency.

In relation to cognitive strategies, the observation particularly focused on the mediation of transcendence, which consisted of seven checklist items. For metacognitive strategies, the analysis concentrated on goal setting, regulation and control of behavior, and shared intention, which collectively support trainees’ abilities in planning, monitoring, and evaluating their reading skills learning activities.

Data were collected using structured classroom observation checklists. Each checklist included items with sub-points categorized as “Yes” or “No” to indicate whether the specific mediation behavior was observed. The frequencies recorded by both observers were averaged, and the results were expressed in percentages to capture the extent of mediation delivered in the lessons.

**Fig 2:**
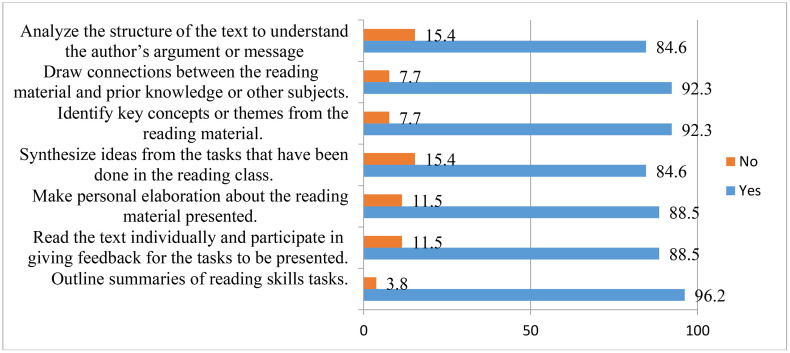
Trainees’ Classroom Practices through Teacher Mediation of Cognitive Strategies.

**Fig 3:**
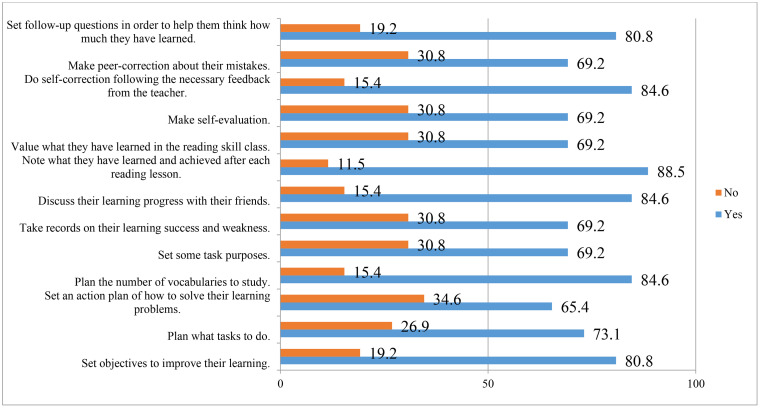
Trainees’ Classroom Practices through Teacher Mediation of Metacognitive Strategies in Reading Lessons.

This analysis thus provides insight into the degree to which teacher educators’ mediation strategies contributed to the development of trainees’ cognitive and metacognitive strategies, ultimately supporting their movement toward autonomous learning. The results of the classroom observations are presented in Figs 2 and 3.

5.7.6.1. **Trainees’ Classroom Practices via Teacher Mediation of Cognitive Strategies in EFL Reading Lessons:** A semi-structured observation was carried out to explore how trainees actually use cognitive strategies to promote learner autonomy by the help of the teacher educator’s mediation of transcendence. This observation, supported by field notes, utilized a checklist containing seven sub-items. The trainees’ classroom activities, guided by the teacher’s mediation, were observed and rated using ‘Yes’ or ‘No’ categories. The frequency of each response was then calculated and converted into percentages to present the findings clearly. In the teaching-learning of reading skills classes, it was observed that teacher-trainees were facilitated/mediated to practice the following classroom activities to develop cognitive strategies:

A semi-structured classroom observation, supported by field notes, was conducted to examine how trainees applied cognitive strategies in reading lessons with the teacher educator’s mediation of transcendence. Using a checklist of seven sub-items, activities were rated in “Yes/No” categories, and frequencies were converted into percentages.

Fig 2 illustrates that trainees were most frequently guided to connect reading materials with prior knowledge (92.3%), identify key concepts during while- and post-reading activities (92.3%), and summarize texts (96.2%). Personal elaboration, individual reading, and peer feedback were also highly practiced (88.5% each). In contrast, synthesizing ideas and analyzing text structure were less frequently supported (84.6% each).

Overall, the findings indicate that mediation of transcendence strongly promoted trainees’ use of cognitive strategies, with summarizing tasks being the most emphasized, while synthesis and text analysis were the least practiced.

5.7.6.2. **Trainees’ Classroom Practices through Teacher Mediation of Metacognitive Strategies in EFL Reading Lessons:** A semi-structured classroom observation, supported by field notes, was conducted to examine how teacher mediation influenced trainees’ use of metacognitive strategies in reading lessons. A checklist with 13 items under planning, monitoring, and evaluating categories was used, and trainees’ activities were rated “Yes” or “No.” Frequencies from both observers were averaged and converted into percentages to present the findings.

A semi-structured classroom observation, supported by field notes, was conducted to examine how teacher trainees applied metacognitive strategies in reading lessons with the support of teacher mediation. The observation checklist included 13 sub-items organized under three categories: planning, monitoring, and evaluating. Trainees’ activities were rated “Yes” or “No,” and the frequencies from both observers were averaged and converted into percentages.

Fig 3 illustrates the findings. The teacher educator consistently encouraged trainees to note their learning achievements after each lesson (88.5%) and to engage in self-correction, peer discussion, and planning vocabulary study (84.6%). Trainees were also guided to set learning objectives, reflect on their progress, plan tasks, and establish task purposes (69.2–80.8%). Less frequently observed activities included making self-evaluations, recording successes and weaknesses, valuing learning outcomes, and setting action plans for problem-solving (65.4–69.2%).

Overall, the results indicate that teacher mediation effectively promoted the use of metacognitive strategies among trainees, with activities related to reflecting on learning outcomes being most frequently practiced, while planning action steps for problem-solving was the least emphasized. The variation in frequencies suggests that certain metacognitive practices were more consistently integrated into classroom routines than others.

### 5.2. Discussion

This study investigated the effects of teacher educators’ mediation on freshman pre-service trainees’ development of cognitive and metacognitive strategies in learning EFL reading skills. It also examined whether such mediation led to significant improvements in trainees’ reading skills achievement. The discussion consolidates key findings, merging repetitive references to learner autonomy and Mediated Learning Experience (MLE). The results provided valuable insights into the impact of teacher mediation on both strategy use and skill development.

#### 5.2.1. Teacher educators’ mediation on trainees’ use of cognitive and metacognitive strategies.

The first research question examined whether teacher educators’ mediation had a statistically significant effect on trainees’ perceived use of cognitive strategies (CS) and metacognitive strategies (MCS) compared to the control group (CG). Paired-sample t-test results for the experimental group (EG) revealed a statistically significant increase in both CS and MCS from pre- to post-intervention, indicating that the teacher educators’ mediation strategies effectively enhanced trainees’ perceived use of these strategies.

In contrast, the CG, which experienced conventional teaching without mediation, did not show significant changes in their perceived use of either strategy. Furthermore, independent-samples t-tests confirmed that the post-intervention differences between the EG and CG were statistically significant, with the EG outperforming the CG in both CS and MCS. Cohen’s d values indicated large effect sizes for the EG, demonstrating a strong impact of teacher mediation, whereas the CG showed only minimal effect sizes.

These findings suggest that teacher educators’ deliberate use of mediation strategies substantially improves trainees’ engagement with cognitive and metacognitive strategies, supporting more autonomous learning in EFL reading skills.

#### 5.2.2. Teacher Educators’ Mediation on Trainees’ Views of the Importance and Mediators’ Use of MLE/Mediation Parameters.

As discussed earlier, teacher educators’ mediation had a significant positive effect on the development of trainees’ perceived use of cognitive and metacognitive strategies (CS and MCS) in reading lessons. To further support this, the experimental group’s (EG) views regarding the importance of mediation parameters and the mediator’s use of these strategies were evaluated post-intervention. Paired-sample *t*-tests and Cohen’s *d* results indicated significant positive changes in the EG participants’ perceptions, confirming the effectiveness of teacher mediation.

Lesson observations corroborated these findings, showing that mediated instruction significantly promoted trainees’ engagement in activities fostering CS and MCS. For cognitive strategies, the highest engagement occurred in outlining summaries, connecting reading materials to prior knowledge, and identifying key concepts or themes, facilitated through the mediation of transcendence. Trainees also demonstrated active participation in individual reading and providing feedback, although analyzing and synthesizing ideas from texts were less frequently practiced.

For metacognitive strategies, trainees engaged positively in activities related to goal setting (planning), regulation and control of behavior (monitoring and evaluation), and shared intention (task purpose setting). These practices reflect the teacher educator’s mediation in supporting trainees’ autonomous learning.

These results align with prior research emphasizing that teacher mediation in the use of learning strategies fosters learner autonomy by promoting control over the learning process, independence, and critical thinking [7,11,19,26,29]. The practical implementation of mediation strategies such as intentionality, reciprocity, and transcendence further supports the development of critical thinking and problem-solving skills, enabling learners to become more independent [6,18,11,34,37]. Additionally, teacher facilitation of metacognitive strategies, including planning, monitoring, and evaluating, has been shown to enhance learner autonomy [20,22,25,27,39].

#### 5.2.3. Teacher Educators’ Mediation on Changes in Trainees’ Reading Skills Performance.

The second research question examined whether teacher-mediated instruction had a statistically significant effect on trainees’ reading skills performance, as influenced by their enhanced use of cognitive and metacognitive strategies (CS and MCS). Results from paired- and independent-samples t-tests indicated that the application of mediation principles and instructional scaffolding significantly improved trainees’ EFL reading skills. Specifically, trainees who received teacher mediation showed significantly higher reading performance compared to those in the control group.

These findings are consistent with previous research [9, 31, 32], which demonstrated that the development of students’ cognitive and metacognitive strategies enhances language performance and academic outcomes. Engaging learners with these strategies promotes better comprehension, improved task performance, and increased learner autonomy. Similarly, students who set their own learning objectives (metacognitive strategies) generally achieve higher performance than those whose goals are externally determined [8, 11].

Moreover, the use of mediation strategies including promoting intentionality and reciprocity, fostering transcendence, and emphasizing meaning contributed to greater student engagement and academic achievement [28, 38, 39].

Overall, the study suggests that teacher-mediated reading instruction effectively improves trainees’ use of CS and MCS, supporting autonomous learning. This enhanced strategic engagement, in turn, translates into measurable gains in reading skills performance and classroom success.

## 6. Conclusions, recommendations, and limitations

This study demonstrates that teacher educators’ mediation significantly enhances EFL trainees’ use of cognitive and metacognitive strategies, thereby fostering learner autonomy in reading skills. By applying Mediated Learning Experience (MLE) principles such as intentionality, reciprocity, and transcendence teacher educators not only improved trainees’ reading performance but also shaped their perceptions of strategy use and promoted greater self-directed learning.

The findings highlight the importance of shifting EFL teacher training programs toward mediation-based, process-focused instruction. EFL curricula should integrate MLE strategies to foster cognitive and metacognitive skills among pre-service teachers. Beyond content delivery, programs should equip trainees with lifelong learning skills, including goal-setting, self-monitoring, reflection, and critical thinking. Integrating MLE strategies into EFL curricula can improve trainees’ reading skills and prepare them to cultivate similar autonomy in their own future students, contributing to broader improvements in teaching and learning outcomes.

Based on these results, it is recommended that English language instructors incorporate mediation strategies into their daily teaching. Such practices can help trainees effectively utilize cognitive and metacognitive strategies, strengthen learner autonomy, and enhance overall language learning outcomes. Freshman students at Kotebe University, and similar learners in other Ethiopian and global contexts, are likely to benefit particularly from mediation-based instruction, especially those struggling to learn reading skills independently.

Potential confounders, such as instructor differences, were minimized by ensuring equivalent qualifications (MA in TEFL) and fidelity monitoring through weekly lesson plan reviews. The Hawthorne effect was reduced by blinding participants to group assignment, and test familiarity was controlled using parallel test versions of similar difficulty. Triangulation of data from reading proficiency tests, questionnaires, and classroom observations mitigated common method bias, ensuring robust findings.

However, this study has certain limitations. The sample size (N = 80) was relatively small, and participants were drawn from a single university in Ethiopia, which may restrict the generalizability of the findings. Additionally, the study focused exclusively on higher education freshman pre-service trainees, leaving unexplored the potential effects of MLE strategies across other grade levels or EFL skill areas. Future research should consider larger, more diverse samples and varied educational contexts to better understand the effectiveness and adaptability of mediation strategies in developing learner autonomy across different EFL learning environments.

Overall, this research underscores the value of learner-centered, mediation-based instruction in EFL teacher training, highlighting structured mediation as a key approach for cultivating autonomous, reflective, and strategically competent educators.

## Supporting information

S1 Data Raw data. (DOCX)
