# Supplementary material for: Enhancing EFL reading proficiency through mediated learning: Impacts on cognitive and metacognitive strategies
Source: PLoS One. 2026 Apr 16;21(4):e0337355. doi: 10.1371/journal.pone.0337355 (PMC13086345; doi:10.1371/journal.pone.0337355)
Supplement: S1 Data — (DOCX) [file pone.0337355.s001.docx]

**Raw Data**

**Appendix A. Pre- and Post- test results of Teacher Trainees in Experimental and Control Group before and after Mediation**

| **Stud. Code** | **Experimental Group** | | **Stud.**  **Code** | **Control Group** | |
| --- | --- | --- | --- | --- | --- |
|  | **Pre-test** | **Post- test** |  | **Pre-test** | **Post- test** |
| SST01 | 16 | 24 | SST41 | 11 | 12 |
| SST02 | 8 | 14 | SST42 | 9 | 11 |
| SST03 | 13 | 18 | SST43 | 14 | 14 |
| SST04 | 11 | 16 | SST44 | 10 | 10 |
| SST05 | 10 | 20 | SST45 | 9 | 9 |
| SST06 | 13 | 17 | SST46 | 12 | 13 |
| SST07 | 17 | 22 | SST47 | 14 | 15 |
| SST08 | 15 | 21 | SST48 | 14 | 14 |
| SST09 | 12 | 18 | SST49 | 10 | 10 |
| SST10 | 11 | 15 | SST50 | 9 | 9 |
| SST11 | 7 | 13 | SST51 | 9 | 8 |
| SST12 | 7 | 14 | SST52 | 7 | 7 |
| SST13 | 8 | 11 | SST53 | 10 | 11 |
| SST14 | 14 | 19 | SST54 | 13 | 13 |
| SST15 | 10 | 15 | SST55 | 9 | 7 |
| SST16 | 12 | 17 | SST56 | 14 | 15 |
| SST17 | 9 | 14 | SST57 | 10 | 12 |
| SST18 | 12 | 16 | SST58 | 16 | 17 |
| SST19 | 14 | 18 | SST59 | 16 | 18 |
| SST20 | 15 | 20 | SST60 | 15 | 16 |
| SST21 | 10 | 15 | SST61 | 11 | 11 |
| SST22 | 8 | 14 | SST62 | 8 | 7 |
| SST23 | 11 | 13 | SST63 | 7 | 8 |
| SST24 | 9 | 11 | SST64 | 7 | 6 |
| SST25 | 8 | 13 | SST65 | 10 | 10 |
| SST26 | 13 | 15 | SST66 | 12 | 12 |
| SST27 | 15 | 18 | SST67 | 12 | 13 |
| SST28 | 13 | 16 | SST68 | 9 | 9 |
| SST29 | 14 | 21 | SST69 | 15 | 15 |
| SST30 | 12 | 15 | SST70 | 11 | 11 |
| SST31 | 7 | 14 | SST71 | 6 | 7 |
| SST32 | 14 | 16 | SST72 | 12 | 12 |
| SST33 | 7 | 8 | SST73 | 8 | 6 |
| SST34 | 10 | 14 | SST74 | 7 | 8 |
| SST35 | 9 | 15 | SST75 | 10 | 9 |
| SST36 | 6 | 9 | SST76 | 5 | 5 |
| SST37 | 12 | 17 | SST77 | 13 | 10 |
| SST38 | 5 | 12 | SST78 | 14 | 19 |
| SST39 | 11 | 19 | SST79 | 13 | 13 |
| SST40 | 9 | 10 | SST80 | 14 | 16 |

**Appendix B. Pre- and Post-mediation results of Experimental Group of Teacher Trainees’ ratings on the importance and Mediator’s use of mediation Parameters/strategies**

| **Stud. Code** | **Pre-Mediation** | | **Stud.**  **Code** | **Post-Mediation** | |
| --- | --- | --- | --- | --- | --- |
|  | **Importance of Mediation Parameters** | **Use of Mediation Parameters** |  | **Importance of Mediation Parameters** | **Use of Mediation Parameters** |
| SST01 | 2.80 | 3.00 | SST01 | 4.00 | 3.70 |
| SST02 | 2.10 | 3.20 | SST02 | 4.00 | 4.40 |
| SST03 | 3.00 | 2.50 | SST03 | 4.60 | 4.30 |
| SST04 | 2.20 | 2.50 | SST04 | 4.20 | 3.80 |
| SST05 | 2.70 | 3.10 | SST05 | 3.60 | 3.50 |
| SST06 | 2.40 | 2.00 | SST06 | 4.20 | 3.90 |
| SST07 | 2.80 | 3.00 | SST07 | 3.80 | 4.20 |
| SST08 | 2.70 | 2.60 | SST08 | 4.50 | 3.70 |
| SST09 | 3.00 | 2.40 | SST09 | 4.30 | 4.00 |
| SST10 | 2.50 | 2.50 | SST10 | 4.20 | 4.10 |
| SST11 | 2.10 | 2.40 | SST11 | 3.70 | 3.70 |
| SST12 | 2.20 | 3.10 | SST12 | 4.70 | 4.70 |
| SST13 | 3.10 | 2.50 | SST13 | 3.50 | 3.60 |
| SST14 | 3.10 | 2.30 | SST14 | 3.90 | 4.30 |
| SST15 | 3.10 | 2.10 | SST15 | 3.90 | 3.90 |
| SST16 | 3.10 | 3.20 | SST16 | 3.80 | 4.20 |
| SST17 | 2.60 | 3.00 | SST17 | 5.00 | 4.60 |
| SST18 | 2.90 | 2.90 | SST18 | 3.50 | 3.90 |
| SST19 | 3.00 | 2.80 | SST19 | 4.00 | 3.90 |
| SST20 | 2.40 | 2.90 | SST20 | 4.00 | 4.20 |
| SST21 | 2.20 | 2.40 | SST21 | 4.20 | 4.40 |
| SST22 | 3.60 | 3.30 | SST22 | 5.00 | 4.50 |
| SST23 | 2.30 | 2.90 | SST23 | 4.60 | 4.10 |
| SST24 | 2.80 | 2.80 | SST24 | 4.10 | 4.00 |
| SST25 | 2.50 | 3.00 | SST25 | 4.00 | 4.20 |
| SST26 | 2.50 | 2.60 | SST26 | 3.60 | 3.80 |
| SST27 | 2.40 | 3.00 | SST27 | 4.20 | 3.70 |
| SST28 | 2.60 | 2.10 | SST28 | 4.10 | 4.10 |
| SST29 | 2.70 | 3.20 | SST29 | 4.10 | 4.20 |
| SST30 | 3.10 | 3.00 | SST30 | 3.20 | 4.00 |
| SST31 | 2.40 | 3.20 | SST31 | 4.50 | 2.90 |
| SST32 | 2.00 | 2.00 | SST32 | 3.50 | 4.10 |
| SST33 | 2.30 | 2.60 | SST33 | 2.60 | 3.60 |
| SST34 | 3.00 | 2.60 | SST34 | 2.80 | 3.30 |
| SST35 | 2.10 | 2.90 | SST35 | 2.90 | 3.50 |
| SST36 | 2.40 | 2.10 | SST36 | 3.20 | 3.90 |
| SST37 | 2.10 | 2.10 | SST37 | 3.00 | 3.00 |
| SST38 | 2.30 | 3.00 | SST38 | 3.20 | 3.60 |
| SST39 | 3.00 | 2.10 | SST39 | 2.90 | 3.70 |
| SST40 | 2.90 | 3.50 | SST40 | 3.90 | 3.20 |

**Appendix C. Pre- and Post-mediation questionnaire results of both Experimental and Control Group Trainees’ ratings on Perceived use of Cognitive and Metacognitive strategies**

| **Stud. Code** | **Experimental Group** | | | | **Stud.**  **Code** | **Control Group** | | | |
| --- | --- | --- | --- | --- | --- | --- | --- | --- | --- |
|  | **Pre-Mediation**  **Results** | | **Post-Mediation**  **Results** | |  |  |  |  |  |
|  |  |  |  |  |  | **Pre-Mediation Results** | | **Post-Mediation Results** | |
|  | **Cognitive**  **Str.** | **Metacog.**  **Str.** | **Cognitive**  **Str.** | **Metacog.**  **Str.** |  | **Cognitive**  **Str.** | **Metacog.**  **Str.** | **Cognitive**  **Str.** | **Metacog.**  **Str.** |
| SST01 | 2.55 | 2.83 | 3.83 | 3.29 | SST41 | 2.00 | 2.07 | 1.50 | 1.71 |
| SST02 | 2.09 | 2.17 | 4.25 | 3.83 | SST42 | 1.73 | 1.78 | 2.00 | 1.67 |
| SST03 | 1.82 | 1.92 | 3.83 | 3.96 | SST43 | 2.09 | 1.96 | 2.50 | 2.38 |
| SST04 | 2.09 | 2.21 | 4.42 | 3.46 | SST44 | 1.45 | 1.57 | 1.58 | 1.71 |
| SST05 | 1.55 | 1.96 | 4.00 | 3.88 | SST45 | 2.00 | 1.43 | 1.92 | 1.92 |
| SST06 | 2.00 | 1.58 | 4.08 | 3.50 | SST46 | 1.64 | 1.83 | 2.42 | 1.58 |
| SST07 | 2.00 | 2.13 | 3.58 | 3.38 | SST47 | 2.36 | 2.13 | 1.58 | 2.21 |
| SST08 | 2.45 | 1.88 | 3.83 | 3.67 | SST48 | 2.09 | 1.48 | 2.17 | 1.92 |
| SST09 | 2.09 | 1.92 | 4.08 | 3.38 | SST49 | 2.00 | 2.21 | 2.67 | 2.00 |
| SST10 | 2.00 | 2.00 | 4.00 | 3.83 | SST50 | 1.82 | 1.61 | 1.58 | 1.67 |
| SST11 | 2.00 | 1.71 | 3.75 | 3.54 | SST51 | 2.00 | 1.70 | 2.17 | 2.21 |
| SST12 | 2.18 | 2.08 | 4.42 | 3.67 | SST52 | 1.64 | 1.39 | 2.42 | 1.79 |
| SST13 | 1.55 | 1.83 | 3.33 | 3.67 | SST53 | 2.45 | 1.83 | 2.00 | 2.00 |
| SST14 | 2.64 | 2.13 | 4.17 | 3.29 | SST54 | 2.09 | 2.04 | 2.75 | 1.75 |
| SST15 | 1.91 | 2.13 | 3.67 | 3.29 | SST55 | 2.00 | 2.17 | 2.50 | 2.08 |
| SST16 | 2.27 | 1.88 | 4.17 | 3.92 | SST56 | 1.91 | 1.74 | 1.50 | 1.63 |
| SST17 | 1.91 | 2.17 | 3.67 | 3.71 | SST57 | 2.27 | 1.86 | 2.25 | 1.71 |
| SST18 | 2.18 | 1.92 | 4.42 | 3.17 | SST58 | 1.55 | 1.74 | 2.92 | 1.50 |
| SST19 | 1.64 | 2.13 | 3.83 | 3.79 | SST59 | 2.27 | 1.96 | 1.17 | 1.75 |
| SST20 | 2.18 | 1.63 | 4.08 | 3.38 | SST60 | 1.73 | 1.44 | 2.25 | 1.96 |
| SST21 | 1.82 | 1.71 | 4.17 | 3.17 | SST61 | 1.64 | 1.96 | 2.67 | 2.13 |
| SST22 | 1.82 | 2.42 | 3.83 | 4.08 | SST62 | 1.91 | 1.43 | 1.50 | 1.63 |
| SST23 | 2.27 | 1.92 | 4.25 | 3.79 | SST63 | 1.64 | 1.62 | 2.42 | 1.88 |
| SST24 | 1.64 | 2.08 | 4.08 | 3.42 | SST64 | 1.82 | 1.92 | 2.17 | 1.67 |
| SST25 | 1.91 | 2.00 | 4.17 | 3.79 | SST65 | 1.73 | 1.61 | 1.75 | 1.63 |
| SST26 | 1.82 | 1.96 | 4.17 | 3.46 | SST66 | 2.00 | 1.57 | 2.25 | 1.83 |
| SST27 | 1.91 | 1.79 | 4.25 | 3.50 | SST67 | 1.64 | 1.65 | 2.83 | 1.75 |
| SST28 | 1.55 | 1.63 | 3.92 | 3.58 | SST68 | 2.09 | 1.78 | 2.00 | 2.08 |
| SST29 | 2.18 | 1.88 | 2.58 | 2.67 | SST69 | 2.73 | 3.09 | 2.50 | 2.50 |
| SST30 | 3.36 | 2.50 | 2.42 | 2.83 | SST70 | 3.18 | 2.65 | 3.08 | 2.92 |
| SST31 | 3.82 | 2.63 | 2.42 | 3.17 | SST71 | 3.18 | 2.48 | 2.33 | 2.00 |
| SST32 | 3.55 | 2.13 | 2.25 | 2.79 | SST72 | 3.27 | 2.52 | 3.08 | 2.58 |
| SST33 | 3.64 | 2.38 | 3.00 | 2.83 | SST73 | 2.91 | 3.09 | 3.42 | 3.17 |
| SST34 | 3.00 | 2.58 | 2.42 | 2.67 | SST74 | 3.00 | 2.82 | 2.83 | 2.71 |
| SST35 | 3.55 | 2.29 | 2.42 | 2.38 | SST75 | 2.73 | 2.74 | 3.17 | 2.88 |
| SST36 | 3.45 | 2.50 | 1.83 | 2.79 | SST76 | 2.91 | 2.48 | 3.50 | 2.58 |
| SST37 | 4.18 | 1.79 | 2.17 | 2.38 | SST77 | 3.82 | 2.48 | 3.00 | 2.38 |
| SST38 | 3.91 | 2.75 | 2.75 | 2.71 | SST78 | 3.09 | 2.35 | 3.08 | 2.21 |
| SST39 | 3.18 | 2.25 | 2.33 | 3.25 | SST79 | 3.27 | 2.18 | 3.08 | 2.58 |
| SST40 | 3.73 | 2.08 | 2.92 | 2.42 | SST80 | 2.82 | 2.13 | 3.67 | 2.21 |

**Data of Classroom Observation collected via Checklists**

**Figure 2.** **Trainees’ Classroom Practices through Teacher Mediation of Cognitive Strategies**

| **No.** | **Observation checklist** | **Yes** | | | **No** | | |
| --- | --- | --- | --- | --- | --- | --- | --- |
| **1.** | **Mediation of transcendence (Cognitive Strategies)** | **Obr.1** | **Obr.2** | **Av. (%)** | **Obr.1** | **Obr.2** | **Av. (%)** |
|  |  | **f(%)** | **f(%)** |  | **f(%)** | **f(%)** |  |
| 1.1. | Analyze the structure of the text to understand the author’s argument or message | 11(84.6) | 11(84.6) | 84.6 | 2(15.4) | 2(15.4) | 15.4 |
| 1.2 | Draw connections between the reading material and prior knowledge or other subjects. | 11(84.6) | 13(100) | 92.3 | 2(15.4) | - | 7.7 |
| 1.3 | Identify key concepts or themes from the reading material. | 12(92.3) | 12(92.3) | 92.3 | 1(7.7) | 1(7.7) | 7.7 |
| 1.4 | Synthesize ideas from the tasks that have been done in the reading class. | 12(92.3) | 10(76.9) | 84.6 | 1(7.7) | 3(23.1) | 15.4 |
| 1.5 | Make personal elaboration about the reading material presented. | 11(84.6) | 12(92.3) | 88.5 | 2(15.4) | 1(7.7) | 11.5 |
| 1.6 | Read the text individually and participate in giving feedback for the tasks to be presented. | 12(92.3) | 11(84.6) | 88.5 | 1(7.7) | 2(15.4) | 11.5 |
| 1.7 | Outline summaries of reading skills tasks. | 13(100) | 12(92.3) | 96.2 | - | 1(7.7) | 3.8 |

**(Total number of observations = thirteen)**

Figure 3: Trainees’ Classroom Practices through Teacher Mediation of Metacognitive Strategies in Reading Lessons

| **2.** | **Mediation of Goal setting; regulation & control of behavior; intentionality/respr (Meta-cognitive strategies)** | **Yes** | | | **No** | | |
| --- | --- | --- | --- | --- | --- | --- | --- |
|  |  | **Obr.1** | **Obr.2** | **Av. (%)** | **Obr.1** | **Obr.2** | **Av. (%)** |
|  |  | **f(%)** | **f(%)** |  | **f(%)** | **f(%)** |  |
| 2.1 | set objectives to improve their learning. | 10(76.9) | 11(84.6) | 80.8 | 3(23.1) | 2(15.4) | 19.2 |
| 2.2 | plan what tasks to do. | 9(69.2) | 10(76.9) | 73.1 | 4(30.8) | 3(23.1) | 26.9 |
| 2.3 | set an action plan of how to solve their learning problems. | 9(69.2) | 8(61.5) | 65.4 | 4(30.8) | 5(38.5) | 34.6 |
| 2.4 | plan the number of vocabularies to study. | 11(84.6) | 11(84.6) | 84.6 | 2(15.4) | 2(15.4) | 15.4 |
| 2.5 | set some task purposes. | 9(69.2) | 9(69.2) | 69.2 | 4(30.8) | 4(30.8) | 30.8 |
| 2.6 | take records on their learning success and weakness. | 8(61.5) | 10(76.9) | 69.2 | 5(38.5) | 3(23.1) | 30.8 |
| 2.7 | discuss their learning progress with their friends. | 10(76.9) | 12(92.3) | 84.6 | 3(23.1) | 1(7.7) | 15.4 |
| 2.8 | note what they have learned and achieved after each reading lesson. | 12(92.3) | 11(84.6) | 88.5 | 1(7.7) | 2(15.4) | 11.5 |
| 2.9 | value what they have learned in the reading skill class. | 9(69.2) | 9(69.2) | 69.2 | 4(30.8) | 4(30.8) | 30.8 |
| 2.10 | make self-evaluation. | 10(76.9) | 8(61.5) | 69.2 | 3(23.1) | 5(38.5) | 30.8 |
| 2.11 | do self-correction following the necessary feedback from the teacher. | 11(84.6) | 11(84.6 | 84.6 | 2(15.4) | 2(15.4) | 15.4 |
| 2.12 | make peer-correction about their mistakes. | 9(69.2) | 9(69.2) | 69.2 | 4(30.8) | 4(30.8) | 30.8 |
| 2.13 | set follow-up questions in order to help them think how much they have learned. | 11(84.6) | 10(76.9) | 80.8 | 2(15.4) | 3(23.1) | 19.2 |

**(Total number of observations = thirteen)**
